# Body Size and Cranial Shape Differentiation in Urban and Rural House Mice (*Mus musculus domesticus*)

**DOI:** 10.1093/iob/obag057

**Published:** 2026-09-24

**Authors:** S C Kupchella, A E Kort, M Phifer-Rixey

**Affiliations:** Department of Biology, Drexel University, Philadelphia, PA 19104, USA; Museum of Paleontology, University of Michigan, Ann Arbor, MI 48109, USA; Department of Biology, Drexel University, Philadelphia, PA 19104, USA

## Abstract

Cities are characterized by elevated temperatures, increased pollution, and high-density human populations which often are accompanied by changes in available resources. These shifts have the potential to drive phenotypic divergence in urban wildlife. Functional morphological traits, such as body size, can mediate interactions between wildlife and habitat and are closely tied to life history and fitness. While examples of functional morphological variation associated with urbanization are increasing, variation in such traits as a response to urbanization remains unexplored for most taxa. Here, we investigated morphological divergence between urban and rural populations of house mice (*Mus musculus domesticus*). House mice are globally distributed in diverse habitats and are a model system with a wealth of phenotypic data, making them useful for the study of the impacts of urbanization on morphology. Using a paired replicate design, we sampled urban and rural populations in three distinct metropolitan regions in the eastern United States. We found that body size was smaller in urban populations. Using 3D geometric morphometrics, we also analyzed variation in cranial shape across habitats. Differences in cranial shape were largely allometric, that is, driven by differences in body size. However, we also uncovered evidence of cranial shape variation between habitats not explained by size. In contrast, we did not find evidence for habitat-driven differences in cranial capacity independent of size. Overall, our results suggest a key role for body size in mediating morphological responses to urbanization and highlight the potential of house mice as a globally distributed model for urbanization.

## Spanish

Las ciudades se caracterizan por sus elevadas temperaturas, altos niveles de contaminación y ser áreas con alta densidad de asentamientos humanos, que frecuentemente van acompañadas de cambios en la disponibilidad de recursos. Estos cambios, pueden influir en la divergencia fenotípica de la fauna urbana. Rasgos morfológicos funcionales, como es el tamaño corporal, puede estar involucrado en las interacciones entre fauna silvestre y hábitat, siendo un rasgo que está cercanamente relacionado con historias de vida y fitness. Mientras que cada vez existen más ejemplos de cómo la variación morfológica funcional está asociada con la urbanización, la variación como un rasgo que responde a la urbanización no ha sido profundamente estudiada para la mayoría de los taxones. En el presente estudio, se investigó la divergencia morfológica entre poblaciones del ratón doméstico (*Mus musculus domesticus*) en áreas urbanas y rurales. El ratón doméstico, es una especie distribuida globalmente a lo largo de diversos hábitats. Asimismo, es un organismo modelo del cual se tiene una basta cantidad de datos fenotípicos, convirtiéndolo en un buen sistema para estudiar los impactos de la urbanización en rasgos morfológicos. A través de un diseño de réplicas pareadas, poblaciones tanto urbanas como rurales fueron colectadas en tres distintas regiones metropolitanas en el este de los Estados Unidos de América. Se observó que el tamaño corporal fue menor en las poblaciones urbanas. Mediante morfometría geométrica 3D, se analizó la variación en la morfología craneal entre los distintos hábitats. Las diferencias observadas en la morfología craneal se deben principalmente a efectos alométricos, promovidos por las diferencias en el tamaño corporal. Sin embargo, se observó variación en la morfología craneal entre hábitats, que no está explicada por el tamaño. Por el contrario, no se encontró evidencia de que el hábitat estuviera asociado con las diferencias en la capacidad craneal, independientemente del tamaño corporal. En conjunto, nuestros resultados sugieren que el tamaño corporal tiene un papel fundamental como mediador morfológico en respuesta a la urbanización, y destaca el potencial que tiene el ratón doméstico como un modelo distribuido globalmente para el estudio de la urbanización.

## Introduction

Urbanization represents a significant and ongoing transformation of natural ecosystems. Globally, urban areas are growing rapidly, with estimates ranging between 0.6 and 1.3 million km^2^ by the year 2050 (Huang et al. 2019). This expansion has altered the structure and function of landscapes and produced novel habitats that differ markedly from the natural areas they replace. Wildlife that persist in cities are subject to a suite of anthropogenic stressors, including pollution (chemical, sound, and light), elevated temperatures, and altered resources such as food and trash, among others. These factors can impact ecological dynamics, changing the ways animals interact with the environment and resulting in phenotypic divergence (Alberti et al. 2017; Thompson et al. 2022). Morphological traits are at the interface between organisms and the environment and thus can shed light on the impacts of urbanization on wildlife. Many such traits (e.g., aspects of body size, cranial, and appendage shape) are closely tied to performance and fitness, and divergence in morphological traits across habitats can signal shifts in function, pointing to potential eco-evolutionary drivers (Arnold 1983). For example, urban crested anoles (*Anolis cristatellus*) have longer limbs and larger toepads than conspecifics from undeveloped sites (Winchell et al. 2018; Howell et al. 2022). While there are performance trade-offs associated with shifts in both traits, longer limbs enhance speed in open spaces and larger toepads improve the ability to cling to smooth surfaces commonly found in urban environments (Winchell et al. 2018; Howell et al. 2022).

Examples of intraspecific morphological differentiation associated with urbanization are growing, with data for a range of vertebrate taxa including birds, reptiles, and mammals (e.g., Giraudeau et al. 2014; Meillère et al. 2015; Winchell et al. 2016; Yu et al. 2017; Winchell et al. 2018; DePasquale et al. 2020; Falvey et al. 2020; Howell et al. 2022; Gangloff et al. 2025; see also Johnson and Munshi-South 2017). In particular, studies have investigated variation in body size. Body size has the potential to influence multiple aspects of a species’ ecology and life history, including thermoregulation, metabolic rate, home range size, predator–prey dynamics, competition, fecundity, and resource acquisition (Kleiber 1932; Blueweiss et al. 1978; Western 1979; Cohen et al. 1993). Among mammals, rodents are the most diverse order and have become important for understanding the impacts of urbanization on biodiversity, as both native and non-native commensal species occupy urban habitats (Castillo et al. 2003; Cavia et al. 2009; Garba et al. 2014; Larson and Sander 2025). Body size differentiation associated with urbanization has now been documented in multiple rodent systems. However, there is no clear consensus on whether cities are associated with larger or smaller body size. Some studies have found that rodents are smaller bodied in urban areas (Wist et al. 2022; Bateman et al. 2023; Ofori et al. 2024). Because of the urban heat island (UHI) effect, such shifts would be consistent with Bergmann’s rule; the observation that body size tends to vary inversely with temperature (Bergmann 1847; Martin and Sheridan 2022). However, smaller body sizes in urban environments may be tied to other ecological drivers, such as predation or reduced resource quality. Other studies have documented body size increases associated with urbanization (e.g., Liro 1985; Pergams and Lawler 2009; Pieniążek et al. 2017; Tranquillo et al. 2024) and highlight potential drivers such as increased resource availability and dispersal ability (Martin and Sheridan 2022). Crucially though, studies vary widely with approaches ranging from surveys of a single species in a single city (e.g., Liro 1985; Wist et al. 2022) to meta-analyses of broad taxonomic groups (e.g., Hantak et al. 2021).

Craniofacial variation associated with urbanization has also been shown in several rodent species (Pergams and Lacy 2008; Pergams and Lawler 2009; Snell-Rood and Wick 2013; Yu et al. 2017; DePasquale et al. 2020; Puckett et al. 2020; Feijó et al. 2025). For example, some studies have reported a trend toward increased cranial capacity in urban environments, purportedly induced by plastic and/or adaptive responses to structural complexity (Snell-Rood and Wick 2013; DePasquale et al. 2020). However, evidence supports the opposite trend in some taxa (Snell-Rood and Wick 2013), and other studies have failed to detect a signal altogether (Puckett et al. 2020). Aside from the brain, the cranial skeleton houses organs used in sensory transduction (e.g., vision, olfaction, and audition) as well as structures critical for resource acquisition and mastication. Dietary ecology is associated with biomechanical changes in loading and bite force capacity that can be detected in the shape of the skull (Maestri et al. 2016; Verde Arregoitia et al. 2017). As such, shape variation in the skull between habitats may indicate potential differences in function, particularly with respect to diet.

Importantly, trait variation can result from changes in body size. Allometry (or allometric variation) is change in a morphological character associated with or explained by differences in size (Huxley 1932; Gould 1966; Klingenberg 2016) which is commonly understood to be a product of developmental integration among traits (Hallgrímsson et al. 2019). Thus, components of morphological variation (i.e., cranial shape variation) can either be mediated by size (allometric), or independent of size (non-allometric) (Klingenberg 2016, Marcy et al. 2024). With respect to cranial shape allometry, predictable and repeatable patterns have been identified in mammals. For example, placental mammals broadly follow patterns of craniofacial evolutionary allometry (CREA), where larger species tend to have relatively longer faces and smaller brain cases (Cardini and Polly 2013; Cardini 2019). Shape changes associated with the CREA pattern have been attributed to the morphological integration between size and masticatory function (Marcy et al. 2024; Mitchell et al. 2024), which may be particularly relevant to the highly specialized diets and feeding structures common among rodents (Samuels 2009; Cox et al. 2012; Marcy et al. 2024).

House mice (*Mus musculus domesticus*) have great potential as a model for studying morphological responses to urbanization. Wild house mice are ubiquitous in urban areas, have short generation times, and their use as a laboratory model has produced an abundance of phenotypic and genetic resources. Here, we investigated morphological differentiation between urban and rural populations of house mice in three metro regions in the eastern United States (New York, NY, Philadelphia, PA, and Richmond, VA). Previous research has shown that mice from these areas are genetically distinct (Phifer-Rixey et al. 2018). Therefore, this paired replicate design allowed us to identify differences among habitats in each region and evaluate to what extent differences are shared among regions, potentially reflecting parallel responses to urbanization (Johnson and Munshi-South 2017; Rivkin et al. 2019). First, we tested for differences in body size, examining both weight and linear size metrics, finding that urban mice are smaller than rural mice. Next, we used 3D geometric morphometrics to quantify and compare the shape of the cranium between habitats. We found significant differences in cranial shape across habitats. Differences were largely allometric, but we also found evidence of cranial shape variation between habitats independent of size. Finally, we tested whether urban and rural populations of house mice differ in their cranial capacity, finding no evidence of differences in cranial capacity beyond those expected due to size. Overall, our results suggest that urban and rural mice differ significantly in body size, a trait with strong ties to fitness via physiology, metabolism, and ecological interactions, and correlated traits such as skull size and shape which also have the potential to impact fitness.

## Materials and methods

### Sampling

House mice were collected using live traps (H.B. Sherman, FL) from three cities in the eastern United States (NYC: New York, NY, PHL: Philadelphia, PA, RVA: Richmond, VA; Fig. 1; Table S1) and the rural areas surrounding them. Trapping areas were defined as urban or rural based on the Global Human Settlement Layer (GHS-SMOD) settlement classification (Schiavina et al. 2023; Pesaresi et al. 2024). Upon collection, mice were euthanized and processed with the approval of the Drexel University Institutional Animal Care and Use Committee (IACUC; Protocol No. LL-122–74) and the Monmouth University IACUC (Protocol No. Asp1802). Standard phenotypic measurements (weight, length, hind-foot length, and tail length) were collected for most specimens. Weight was measured using a digital scale (Ohaus Scout SPX421, ±0.003 g), and length measurements were recorded with a ruler (±0.5 mm). For a subset of specimens, skins, skeletons, and skulls were prepared and deposited at the Yale Peabody Museum in New Haven, CT, USA (Table S1). After processing, we used digital calipers (TEC Industrial) to obtain length measurements of the left femur from the superior surface of the femoral head to the inferior surface of the medial condyle (±0.03 mm). The mediolateral width of each femoral shaft was also collected at the midpoint of the diaphysis. Previous research has demonstrated near-perfect bilateral symmetry of long bone features in house mice (Margolis et al. 2004; Beaucage et al. 2016). Therefore for specimens with broken left femora, we recorded contralateral measurements on the right femur. Femoral length measurements were recorded three times, and the average of the three was retained for analysis.

**Fig. 1:**
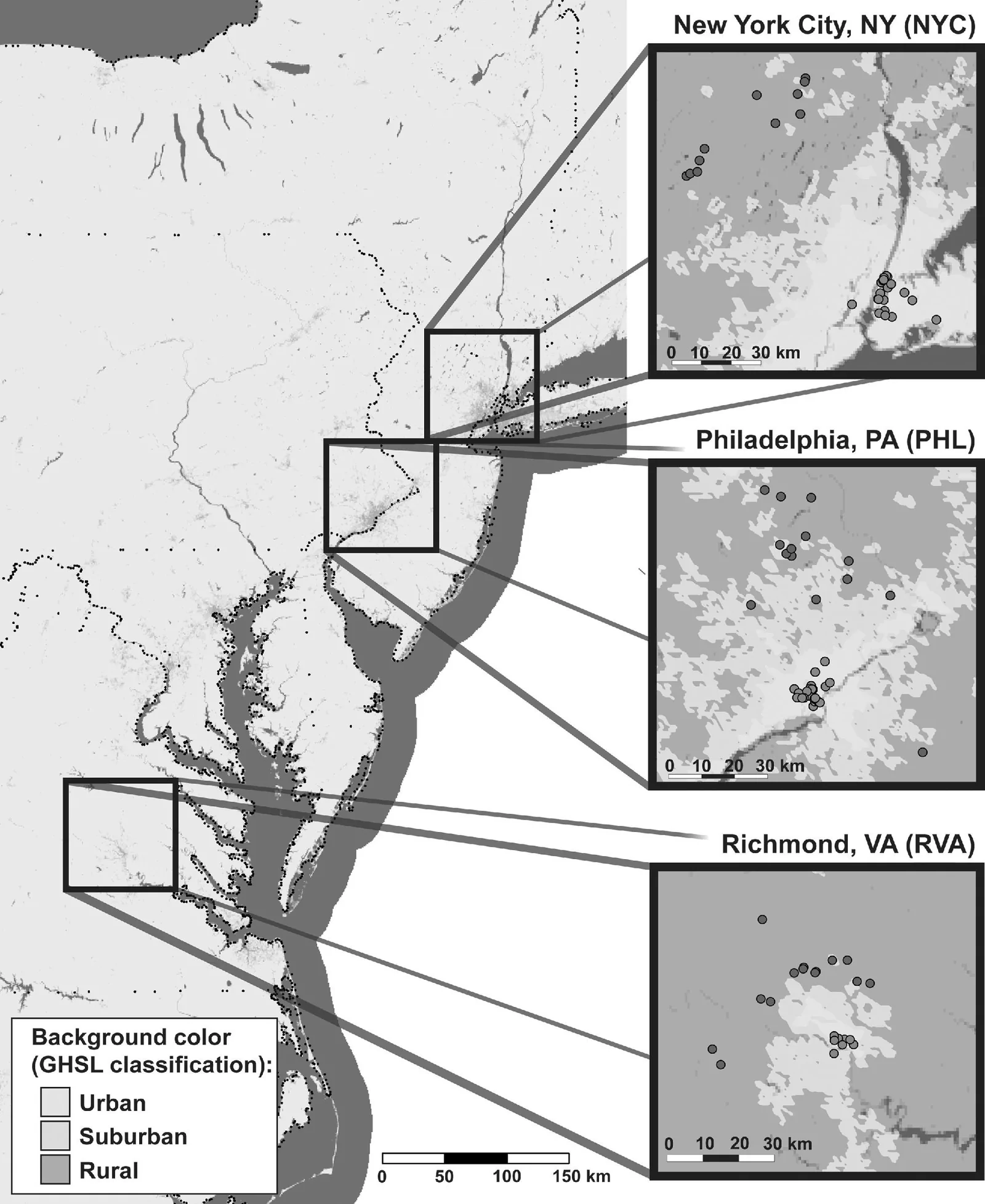
*Map of sampling sites.* House mice were collected from highly developed and less developed sites in three metro regions: New York City, NY., Philadelphia, PA., and Richmond, VA. Inset background color indicates degree of urbanization [GHSL-SMOD (Schiavina et al. 2023; Pesaresi et al. 2024)]. Within insets, orange points indicate urban sites and blue points indicate rural sites. Figure adapted from Giancarli et al. (2026).

Mice used in this analysis were collected from June 2022 to March 2025. To minimize the likelihood of including related individuals in the datasets, we prioritized collection from structures and properties that were >500 m apart. When working in a new habitat for a taxon, there are many open questions about population genetic structure. However, even in rural areas where physical barriers to movement are sparse, house mice typically do not effectively disperse and live in semi-related demes (Pocock et al. 2004, 2005). The literature suggests that for small mammals, urban areas should present many barriers to movement and gene flow (Munshi-South and Kharchenko 2010; Miles et al. 2019; Stevens et al. 2025). Therefore, we chose 500 m as a conservative measure that would minimize relatedness in both habitats even though it made urban sampling much more challenging. Accordingly, if more than one mouse was collected from the same location, only one was retained in the analysis with the decision based on balancing the collection of data for both sexes.

It is not possible to obtain accurate age estimates for wild-caught specimens. As a result, we used a general developmental indicator (hindfoot length ≥18mm) to categorize samples as adult, removing all individuals with lower values with three exceptions. Two specimens had no recorded hindfoot measurement but were otherwise typical for adults and thus were included. In addition, one specimen with a hindfoot measurement of 17 mm was retained based on body weight which clearly placed it as an adult (19.62 g vs. 15.71 g [average]; Table S1). Additionally, multiple pregnant females included in the initial collection were omitted from body weight analyses. Because of these considerations, and because some traits could not be collected for all individuals due to damage, we created three distinct datasets for each of the major morphological analyses: weight (*n* = 80), linear size traits (*n* = 81), and cranial traits (*n* = 72).

### Body mass and linear size metrics

We analyzed multiple aspects of body size, including body mass, body condition, body length, femur length, and femoral mediolateral width by fitting GLMs in RStudio (R Core Team 2024; Posit team 2025). We investigated body mass, body condition, and body length metrics to determine overall size (Fig. S1). Femoral length was chosen as a proxy for body length (Melin et al. 2005), with femoral mediolateral width serving as an indicator of potential differences in mechanical loading and/or locomotor ability (Wallace et al. 2012; Castro and Garland Jr. 2018). Model assumptions were diagnostically tested using the Shapiro–Wilk test for normality and Levene’s test for homogeneity of variance where appropriate. To test for significant relationships, Type II analyses of variance (ANOVAs) were implemented in the *car* package (Fox and Weisberg 2019). All models were fit with three primary predictors: sex, habitat (urban/rural), and metro region (NYC, PHL, and RVA). Where relevant, additional models were created with size metrics included as a covariate. For example, to evaluate the contribution of habitat to differences in weight, we included body length as a covariate to account for scaling. For all analyses, α < 0.05 was used to determine significance. We further analyzed pairwise comparisons using post-hoc tests in the *emmeans* package (Lenth 2025) to assess significant interaction terms and obtain estimated marginal means (EMMs) and differences in EMMs among groups.

### Cranial morphology

#### Micro-computed tomography

We used a Scanco Medical µCT 45 instrument (Scanco Medical, Brüttisellen, Switzerland) housed at the Penn Center for Musculoskeletal Disorders (University of Pennsylvania) to generate micro-CT image data for each available skull. Up to six mouse skulls were stacked 3 × 2 and packed with polyethylene foam into a 50 mL conical vial to prevent movement during scanning. Specimens were scanned at 12 µm resolution (55 kVp, 8 W, 300 ms). Using the scanning software, segments of the tube were isolated such that three skulls were scanned concurrently, resulting in two sets of image stacks per vial.

#### Landmarking

3D digital renderings of mouse crania were generated in 3D Slicer (Kikinis et al. 2013). Of the mice collected, 95 adult crania were scanned. Of these, 16 with fractured or missing elements and seven from duplicate locations were excluded, leaving 72 skulls for comparative morphometric analysis. Each skull was digitized using a fixed-landmark scheme adapted from Pallares et al. (2014,^ 2016^). This scheme was selected for its broad anatomical coverage and potential to capture variation in overall cranial shape. Some changes to the scheme were made. One landmark that was difficult to reliably digitize was replaced with two points ([RLP, LLP: 2, 3] see below) to capture variation in the width of the brain case. Additionally, two points on each zygomatic arch were dropped and replaced with 20-point semi-landmark curves to increase the density of points on a structure that lacks homologous landmarks (Table S2, Fig. S1).

Due to the bilateral symmetry of the cranium, it is common to landmark one half of the structure and reflect points across the midsagittal plane. This approach was implemented to reduce placement error and limit asymmetric variance in the dataset. To achieve this, we first extracted and manually digitized landmarks that defined the midsagittal plane. We used a custom script executed in the 3D Slicer python terminal to create a plane of symmetry and reflect manually digitized right lateral points (RLP) and right zygomatic curves across the plane, producing a mirrored left lateral point (LLP) and left zygomatic curve point list (Table S2, Fig. S1). The five point lists were then merged using the *Merge Markups* utility in Slicermorph (Rolfe et al. 2021) to produce an 86-point data set capturing the complete shape configuration of each mouse skull. On a significant number of the skulls, one or both pterygoid processes were poorly rendered, reflecting incomplete capture during scanning. Thus, prior to testing, two landmarks originally digitized on the processes were removed, resulting in a final dataset of 84 landmarks as shown in Supplementary Tables S1, S2, and Supplementary Fig. S1.

#### Endocasts

Endocranial segments of each skull were generated using the *Segment Endocranium* utility in Slicermorph with a smoothing kernel size of 2.00 mm and maximum hole size of 4.00 mm. The resulting cranial endocasts were visually inspected to ensure that regions rendered appropriately, and that no artifacts were present. If the first run of the software produced an endocast with irregular projections or artifacts emanating from the surface of the segment, then the software was run again with the same parameters, and data were only retained if artifacts were resolved following the second iteration. Volumetric data was collected for each endocast in the *Segment Statistics* module.

#### Geometric morphometrics

To test the effects of sex, habitat, and metro region on cranial shape, raw landmark coordinates were exported to RStudio. We first implemented a Generalized Procrustes Analysis (GPA) (Rohlf and Slice 1990), in the *geomorph* package (Baken et al. 2021; Adams et al. 2025) using a generalized least squares criterion to remove rotation, translation, and scale. The GPA with partial Procrustes superimposition was performed on 48 fixed and 36 sliding semi-landmarks. The Procrustes aligned coordinates were then tested against the set of categorical variables with a permutation ANOVA set to 1000 iterations using the “procD.lm” function. The centroid size, calculated as the square root of the sum of squared distances of the landmark configurations from their geometric mean, was retained and added as a covariate to control for potential size and shape covariation.

We then extracted major axes of shape variation by performing a principal component analysis (PCA) using the “gr.prcomp” function in *geomorph.* The highly dimensional nature of 3D coordinate data results in many defined principal components (PCs) that represent unstructured noise or fine-scale local variation. To identify PCs that correspond to major biological patterns of shape variation in the dataset, we performed a parallel analysis using Horn’s test of principal components with the *paran* package (Dinno 2009). This technique compares observed eigenvalues to those generated from a randomly permuted null distribution, and only components whose eigenvalues exceeded the simulated threshold were retained for further analysis. We then used these PCs as response variables to test the effects of the predictors on shape while removing redundancy with multivariate analysis of covariance (MANCOVA). To ensure that the aforementioned reflection technique did not bias results, we also performed each of the above tests (procD.lm, MANCOVA) on only the manually digitized right-side landmarks. For all analyses, α < 0.05 was used to determine significance.

#### Allometry

We tested and visualized two allometry models using the “plotAllometry” function to understand potential differences in the allometric slope and intercept between habitats. Models were fit with an interaction term (shape ~ size * habitat) as well as an additive term (shape ~ size + habitat) (Fig. S3). We then identified the common allometric vector and decomposed shape into allometric and non-allometric components using the “calculate common allometric component” (CAC) function in *Morpho* (Schlager 2017). Consistent with the Gould–Mosimann framework for allometric accounting in geometric morphometrics (Klingenberg 2016), this method performs a multivariate regression of shape on centroid size to extract residual shape components (RSCs) that represent size-independent shape variation. We visualized allometric relationships by plotting centroid and other size variables against PC1 (Fig. 3; Fig. S4A–D). To validate the assumption that PC1 represented allometric shape change, we directly compared PC1 scores with CAC scores using a correlation test (Fig. S3).

#### Size-mediated and size-independent visualizations

We visualized patterns of cranial shape variation by calculating mean shapes from the Procrustes aligned coordinates for each habitat. We used the “findMeanSpec” function in *geomorph* to identify the specimen in the dataset whose landmark coordinates were closest to the consensus mean configuration established by the GPA and used it as the reference specimen for all visualizations (Fig. 4). A 3D mesh of the reference specimen was first warped to the overall mean and then plotted to the mean configurations for each group. The “meshDist” function was used to create heatmaps with color representing mesh-to-mesh Euclidean distances of each group from the sample mean. To visualize patterns of shape variation between urban and rural environments in a size-free shape space, we back-projected the RSCs into Procrustes space and again calculated group means for the size-corrected data (Fig. 4).

## Results

### Body size

#### Urban mice weigh less than rural mice

Habitat significantly contributed to variation in body weight analyses with [*P* = 0.007] and without [*P* = 0.015] body length as a covariate (Table S6). Across both models, all other factors and interaction terms were not significant, except for body length in the model where it was a covariate [*P* < 0.001]. EMMs comparisons of the covariate model revealed a difference in weight of 1.74 g [*P* = 0.029] between urban (14.4 g) and rural (16.1 g) populations, suggesting that, even when accounting for body length, urban mice weigh ~10.8% less on average than rural mice (Fig. 2A). Here and for all subsequent linear models, assumptions of normality and homogeneity of variance were met (Shapiro–Wilk, Levene’s test *P* > 0.05).

**Fig. 2:**
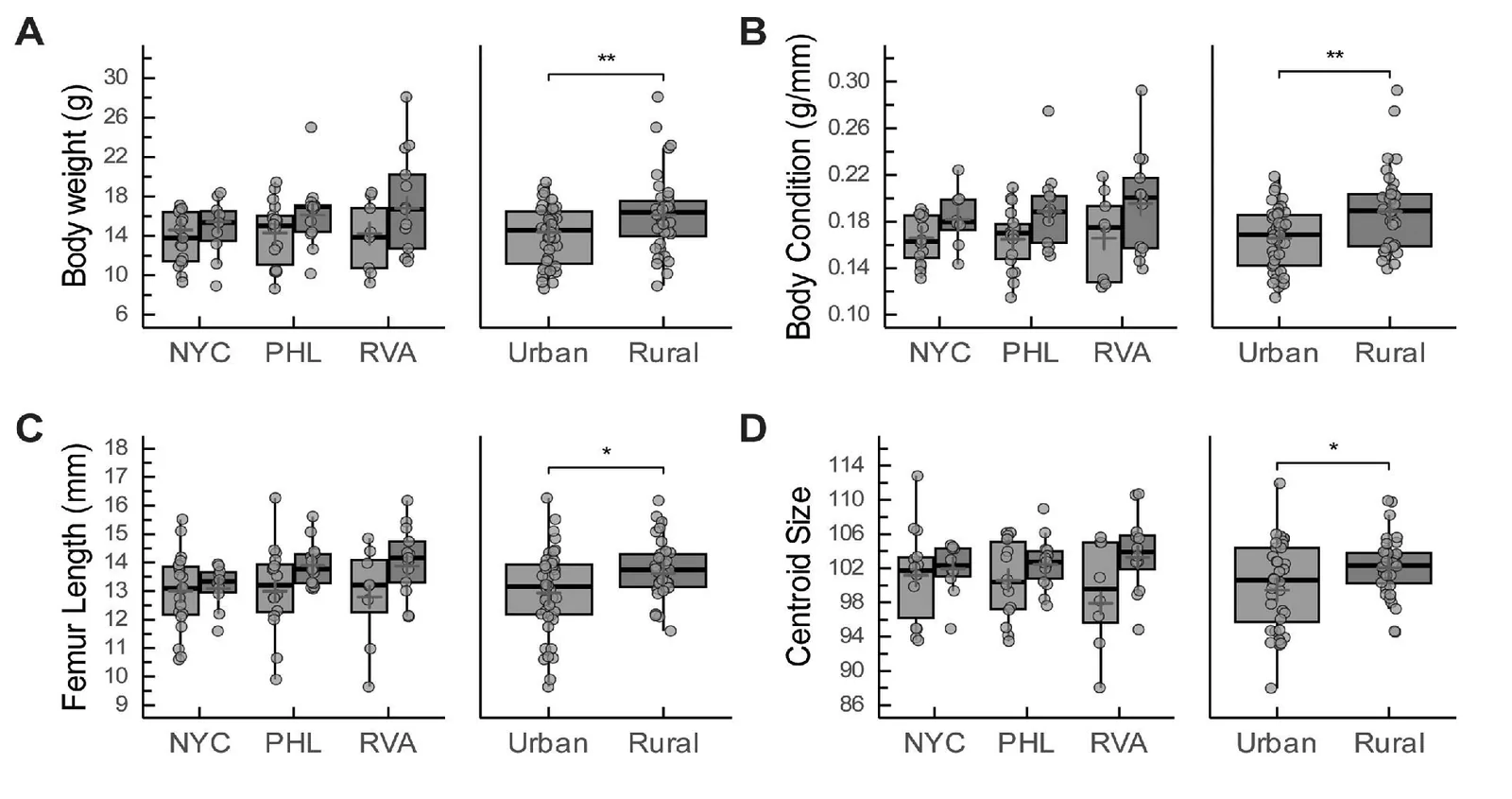
*Urban mice are smaller than rural mice across several key metrics*. Boxplots are shown for **(A)** weight, **(B)** body condition, **(C)** femur length, and **(D)** cranial centroid size within each habitat and metro region and aggregated over metro regions (*P < 0.05, ^**^P < 0.01). (**+**) symbols indicate the EMM for each group when averaged over other factors as predicted by *emmeans*.

We also considered body condition (body weight divided by length). Results of this analysis were similar, with habitat contributing significantly to variation [*P* = 0.003] but not additional factors or interaction terms. Contrasts implemented in *emmeans* predicted a 0.023 g/mm difference in EMMs of urban and rural populations [*P* = 0.015] with body condition of urban mice estimated as ~12.2% lower than their rural conspecifics. Overall, these results suggest that urban mice weigh less and are lighter per unit length compared to rural mice (Fig. 2A, B).

#### Urban mice are smaller than rural mice

We analyzed body length, femur length, and femoral mediolateral width to assess linear size variation. First, we considered body length and found that no factors or interactions including habitat [*P* = 0.114] contributed significantly to variation. However, this metric can be noisy because it is typically calculated by measuring the total length of the specimen (from tip to tail) and subtracting a second measure for the tail (Jewell and Fullagar 1966; Di Masso et al. 1991). As a result, there are two opportunities for error. In addition, it is collected in the field using a ruler with lower precision than digital calipers. Therefore, we also analyzed femur length, which is collected in the lab after specimen preparation as a proxy for overall body length (Melin et al. 2005). Body length and femur length were significantly correlated [*r* = 0.71, *P* < 0.001]. We found that habitat, but no other factors or interactions, contributed significantly to variation in femur length [*P* = 0.015], indicating a reduction in overall body size in the urban populations (Fig. 2C).

The effect of habitat on femoral mediolateral width [*P* = 0.854] was not significant in a model tested without femur length included as a covariate, though sex was a significant predictor [*P* = 0.041). However, in the covariate model, both habitat [*P* = 0.019] and sex [*P* < 0.001] were significant. Femur length also significantly predicted femoral mediolateral width [*P* < 0.001]. With results averaged over the other factors, EMMs contrasts revealed that rural mice (estimate: −0.055 mm, *P* = 0.125) and females (estimate: −0.103 mm, *P* = 0.004) had more gracile (i.e., thinner) femora than urban mice and males, respectively. Overall, these findings provide evidence for linear size differences between urban and rural mice, with urban mice on average having shorter and more robust femora than their rural counterparts, an indication of reduced body size.

### Cranial size and shape

#### Consistent with body size, urban mice have smaller crania

Habitat was a significant predictor of centroid size [*P* = 0.035], but not when tested in a model with body length included as a covariate [*P* = 0.178]. In the covariate model, body length significantly predicted centroid size [*P* < 0.001]. No other factors or interaction terms were significant. EMMs comparisons revealed that, consistent with linear size metrics, urban mice (estimate: 99.9) have lower EMM centroid sizes (*P* = 0.043) than their rural counterparts (estimate: 102.6) (Fig. 2D). Thus, cranial size differs among habitats, but that difference is explained by differences in body size.

#### Cranial shape variation is largely explained by allometry

First, we tested whether habitat, metro region, sex, or size contributed to variation in cranial shape. Procrustes ANOVA with the aligned coordinates as a response variable revealed that centroid size was the top predictor of cranial shape in the full model [*Rsq* = 13.1%, *P* = 0.001]. With centroid included as a covariate, habitat [*Rsq* = 2.3%, *P* = 0.02] and metro region [*Rsq* 3.8%, *P* = 0.009] also contributed significantly to variation. Sex and associated interaction terms were not significant (Table S7). Choice of dataset, (reflected vs. the manually digitized right-side landmarks) did not affect the results of the Procrustes ANOVA (Table S7).

We also considered a similar model via MANCOVA using the PCA to summarize variation in shape. Parallel analysis of the PCs indicated that the first 9 PCs captured meaningful shape variation (Table S5, Fig. S2) and these PCs were retained (PC9 proportion of variance = 2.8%; cumulative proportion of variance = 66.4%, Tables S3 and S4). The first three PCs (PC1-3) accounted for 19.9%, 10.8% and 10.7% of the variation, respectively (Table S4). MANCOVA results were concordant with the findings from the Procrustes ANOVA, with centroid size, habitat, and metro region [all *P* < 0.001] identified as significant predictors (Table S8). There were no appreciable differences between results of the MANCOVA from the reflected and manually digitized set of landmarks (Table S8).

The significant contribution of centroid size to shape variation suggested that allometric scaling effects may be important in this system. We tested a reduced allometric model and found that the (log)centroid:habitat interaction term was not significant (Fig. S3), indicating that urban and rural mice occupy a shared allometric slope. Therefore, we visualized shared shape ~ size covariation by plotting centroid on the first PC axis (Fig. 3B). We verified that PC1 captured the size-related shape variation by directly comparing CAC scores with PC1 scores (Pearson’s *r* = 0.99, Fig. S3). This strong association suggests that the primary axis of shape variation adequately captures the allometric signal, and that PC1 can be effectively treated as the allometry axis.

**Fig. 3:**
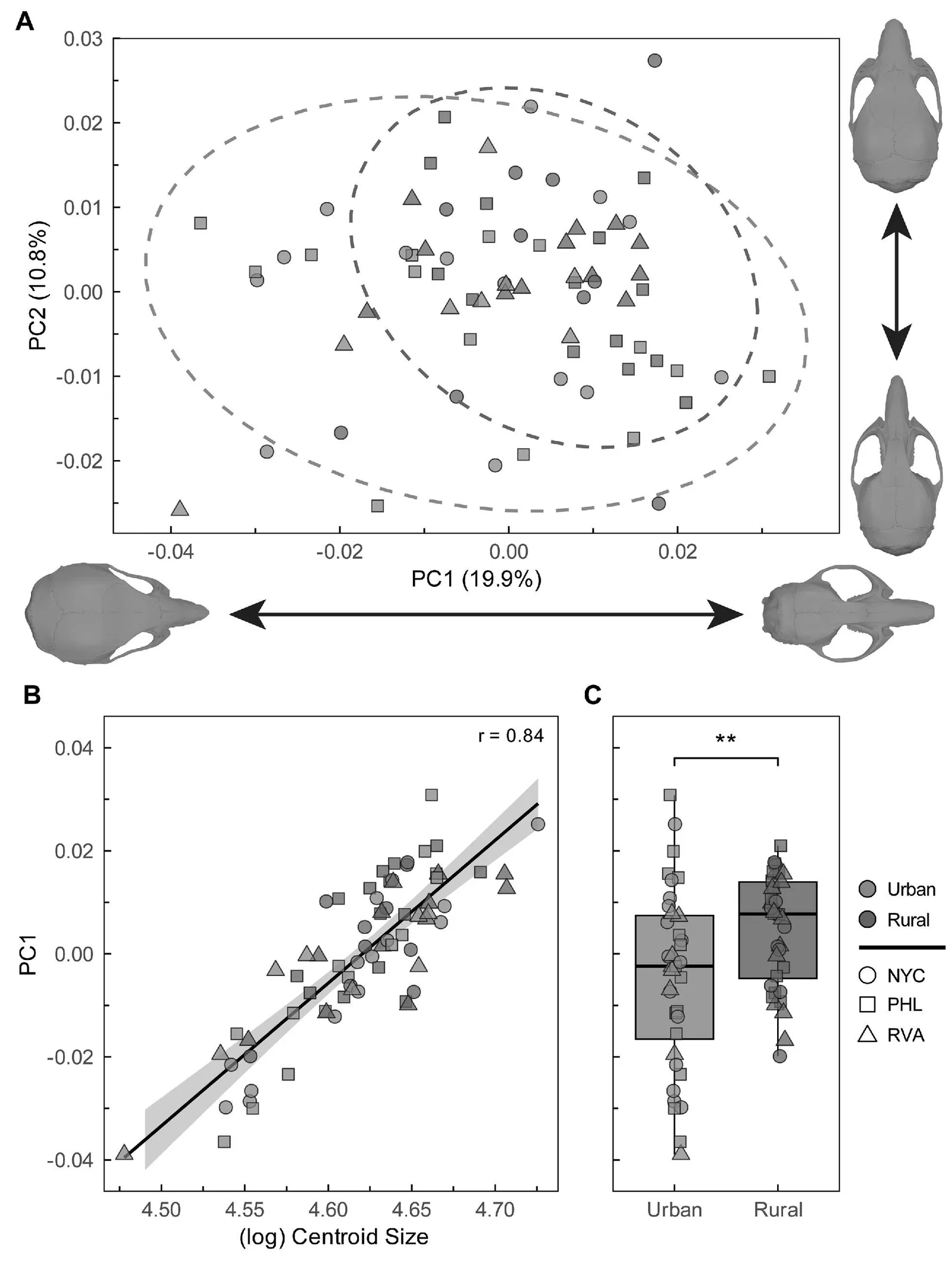
*Allometry drives differences in cranial shape across habitat.*  **(A)** PCA plot of the first two principal components accounting for ~30% of the overall variation in cranial shape. PC1 is the allometric shape axis. Exaggerated cranial warps are depicted to demonstrate shape changes associated with the extremes of each axis. **(B)** The strong positive linear relationship between (log) Centroid Size andPC1 indicates an allometric scaling effect (shaded band = 95% CI of the fitted linear regression). **(C)** PC1, which captures cranial shape differentiation driven by size, varies across habitat (^**^P < 0.01). Boxplots show the distribution of PC1 scores within urban and rural habitats.

The positive linear relationship between centroid and PC1 indicates that skull shape changes systematically with size, with larger crania associated with positive PC1 scores and smaller crania associated with negative PC1 scores (Fig. 3B). Differential placement along the shared vector suggests that urban and rural mice occupy distinct regions of cranial morphospace according to their size (Welch’s two-sample *t*-test [*P* = 0.009], Fig. 3C). We plotted other body size metrics against PC1 to visualize their association with cranial shape (Fig. S4A–D). These variables also showed a positive linear relationship with PC1, suggesting that the primary axis of cranial shape variation reflects general body size allometry and not scaling patterns specific to the size of the skull. Shape differences along PC1 are associated with changes in the length of the rostrum, width and angle of the zygomatic region, and globularity of the brain case (Fig. 3A).

Visual comparison of habitat mean shapes suggests that the skulls of urban mice are horizontally compressed (anteroposteriorly shorter) and vertically expanded (dorsoventrally taller) relative to rural mice (Fig. 4A,C,E,G). In comparison, rural crania are longer along the anteroposterior axis, with a flatter and wider appearance. Additionally, restriction along the anteroposterior axis in the urban population suggests shorter molar tooth rows, and a relatively shorter distance between the tooth rows and the most anterior aspect of the rostrum. Conversely, rural crania show an increase in this distance, resulting in longer rostra when compared to the urban mean.

**Fig. 4:**
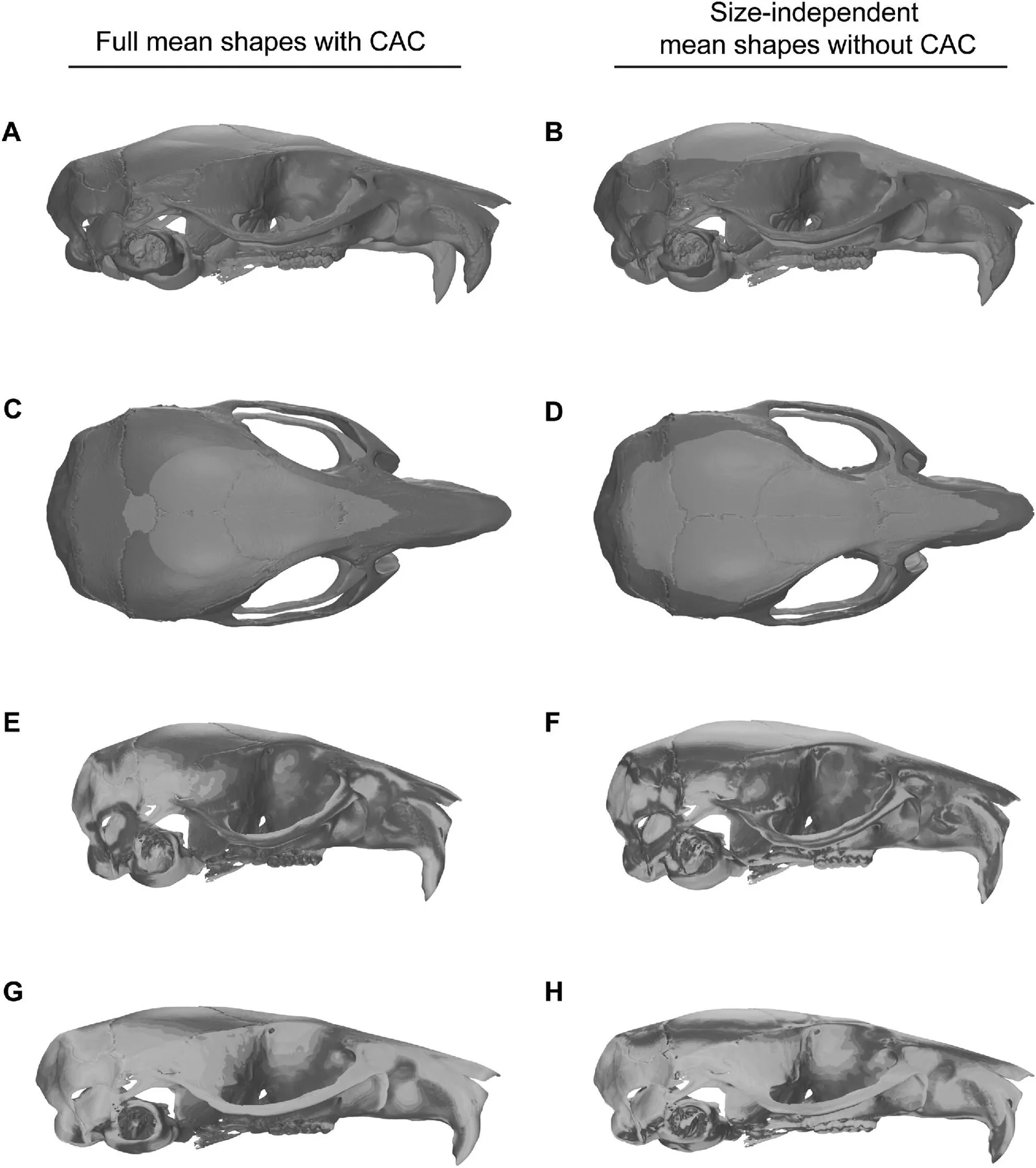
*Urban and rural mice differ in cranial shape with variation both mediated by size (left column) and independent of size (right column).* Mean shapes are shown with **[A, C, E, G]** and without **[B, D, F, H]** the common allometric component (CAC) for the habitat variable. Panels A-D show overlapping mean shapes in both lateral **(A, B)** and dorsal **(C, D)** views for comparison. Colors in panels **[A, B, C, D]** differentiate habitat (orange = urban, blue = rural). Panels E–H show urban **(E, F)** and rural **(G, H)** heatmaps, with warmer colors indicating regions of greater distance from the mean, and cooler colors indicating similarity. Shape differences are magnified 20×. Skulls in panel **(C)** were symmetrized for visual clarity. Unedited meshes are provided in Supplementary Fig. S5.

#### Urban and rural mice differ in cranial shape after accounting for size

The significant effect of habitat in both the full and reduced allometry models indicates a shift of the slope resulting in different intercepts between the populations (Fig. S3). To interpret size-independent cranial shape differences associated with the intercept shift between habitats, we extracted the CAC from the shape data and again generated an overall mean configuration from the global population. This configuration was then warped to the population means to visualize habitat-specific patterns of skull shape (Fig. 4B,D,F,H). Size-independent mean shape comparisons show a reduction in the magnitude of difference between urban and rural shapes along the anteroposterior axis. Additionally, urban crania appear even more vertically expanded, with dorsal and ventral surfaces visible beyond the rural mesh (Fig. 4B and D). The vertically taller and deeper skull also results in a comparatively greater slope extending from the cranial vault to the rostrum in the urban population.

### Cranial endocast volume

#### Variation in cranial endocast volume is driven by body size

Habitat did not significantly contribute to variation in endocast volume in models with [*P* = 0.849] or without [*P* = 0.078] cranial centroid size included as a covariate. Interactions between metro region and habitat [*P* = 0.047] and sex and habitat [*P* = 0.032] were marginally significant only in the model without centroid size as a covariate. However, centroid size was a significant predictor when included as a covariate [*P* < 0.001; Table S6] and volume and cranial centroid size were highly correlated (Fig. S4). Overall, results suggest that variation in cranial endocast volume is driven by variation in body size. Differences in cranial volume between habitats are consistent with what would be predicted given differences in size. Therefore, there is no evidence for house mice having relatively greater endocranial volume in urban environments.

## Discussion

We investigated morphological differentiation between urban and rural populations of *M. m domesticus* across three US cities and found significant variation in multiple traits. Urban mice weigh less overall and per unit length compared to their rural conspecifics. In addition, urban mice have shorter femora (a proxy for body length) and smaller cranial centroid sizes (a proxy for skull size). Together, these results indicate that urban mice are smaller bodied overall and also weigh less for their body size. Our analysis of cranial shape revealed strong covariance between size and shape, with centroid size accounting for ~13% of the variation in the permutation model. Metro region (3.8%) and habitat (2.3%) accounted for less variation but still emerged as significant predictors. These findings point to a clear signal of allometry, with most of the cranial shape variation attributable to size, but some allometry-free variation attributable directly to habitat and metro region. Importantly, however, urban and rural mice differ in size, meaning that the size-related shape variation itself can be interpreted indirectly as a habitat effect.

### Potential drivers of body size differentiation in response to urbanization

Here, we found that cities were associated with reduced body size and condition. Meta-analyses of body size variation in response to urbanization suggest that overall, rodents tend to be larger in urban environments (Santini et al. 2019; Hantak et al. 2021). Nevertheless, results from studies of individual rodent taxa are mixed. Some, like ours, have found that body size metrics are negatively correlated with urbanization class or relevant proxies (i.e., human population density), including body length [*Peromyscus maniculatus* (Guralnick et al. 2020); *Peromyscus leucopus* and *Tamias striatus* (Lopez 2024)], body mass [*Sciurus vulgaris* (Wist et al. 2022); *Dipodomys merriami* (Bateman et al. 2023); *Mus musculoides* (Ofori et al. 2024; Biswal et al. 2025); *Mastomys natalensis* (Biswal et al. 2025)], and body condition [*Sciurus vulgaris* (Wist et al. 2022); *Chaetodipus* spp., *Perognathus* spp., and *Dipodomys merriami* (Bateman et al. 2023); *Gerbilliscus kempi* (Ofori et al. 2024); *Mastomys natalensis* and *Mus musculoides* (Ofori et al. 2024; Biswal et al. 2025)]. On the other hand, studies have identified increases in aspects of body size associated with urbanization [e.g., *Apodemus agrarius* (Liro 1985; Pieniążek et al. 2017); *Sciurus carolinensis* (Tranquillo et al. 2024)].

The lack of consistency across studies suggests that the factors driving variation in body size are likely diverse and the effects of those factors are also likely mediated by diverse life histories within the very speciose Rodentia. Body size is an important functional trait tied to multiple aspects of a species’ ecology and life history, including metabolic rate, thermoregulatory ability, diet, predation, vagility, and fecundity, among others (Kleiber 1932; Blueweiss et al. 1978; Western 1979; Cohen et al. 1993). There are many factors, both abiotic and biotic, associated with urbanization that may impact body size. For example, physiological constraints imposed by body size may create energetic challenges in response to the thermal environment. These thermoregulatory and energetic dynamics are thought to underlie Bergmann’s rule; the observation of clinal patterns of body size variation where endotherms are larger in colder environments and smaller in warmer ones (Bergmann 1847). Size variation consistent with Bergmann’s rule has been repeatedly documented in mammalian taxa (Meiri and Dayan 2003; Blackburn and Hawkins 2004), including in house mice (Lynch 1992; Phifer-Rixey et al. 2018). The application of Bergmann’s rule in the context of UHI predicts smaller body sizes in comparatively warmer urban environments and has been a recent focus in the urban ecology literature (Merckx et al. 2018; Hantak et al. 2021; Bateman et al. 2023).

However, there are many other factors to consider, including diet, predation, and dispersal/range size, that may contribute to body size variation across urban and non-urban habitats. Dietary shifts between habitats can occur as urban populations exploit more anthropogenic resources, and the quantity and quality of resources available to urban populations can either increase or decrease body size (McCleery 2015; Martin and Sheridan 2022). Stable isotope analyses in these *M. m. domesticus* populations uncovered diets higher in animal protein in cities along with associated changes in gut microbial composition (Giancarli et al. 2026). Rural mice are expected to be primarily dependent on plant-based animal feed while urban mice likely have greater access to human-derived food sources and protein-based companion animal feed.

Urbanization can also alter predation and has been shown to result in an increase in predator density, but an overall decrease in predation pressure; “the predation paradox” (Fischer et al. 2012). This release from predation may predict larger body size in small taxa. However, this may not be the case for commensal species. Commensals such as house mice are likely more susceptible to direct encounters with domesticated animal predators and humans employing lethal pest management practices which can result in high mortality (Pocock et al. 2004). It is, therefore, plausible that a reduction in body size may reduce detection and ultimately predation.

Dispersal ability and range size are positively correlated with body size (Lindstedt et al. 1986; Sutherland et al. 2000; Santini et al. 2013; Martin and Sheridan 2022). In urban habitats, species with foraging habits or reproductive strategies that require large ranges and/or dispersal may experience pressure for enhanced vagility, especially if habitat patches are unevenly distributed or fragmented across the landscape. This hypothesis likely has little relevance for house mice which are not expected to disperse over large distances without human assistance (Mikesic and Drickamer 1992; Pocock et al. 2005), but other rodents have relatively large home ranges and can traverse large distances despite structural barriers in urban habitats (Tolkachev 2016; Dammhahn et al. 2020).

Finally, in this study and others, genetic differentiation likely contributes to phenotypic differences across habitats. The traits investigated here have a complex genetic basis (e.g., MacArthur 1949; Cheverud et al. 1996; Kenney-Hunt et al. 2006; Chan et al. 2012; Pallares et al. 2015; Parmenter et al. 2016) and are influenced by both the environment and genetic variation. We have employed closely-paired sampling to minimize the confounding effects of genetic structure and environmental factors unrelated to habitat (e.g., climate) that may impact trait variation. Nevertheless, it is likely that there is genetic differentiation among habitats within metro regions driven by evolutionary processes like gene flow and drift. Whatever the underlying processes, it is clear that the effect of urbanization on body size can be heterogeneous (Callaghan et al. 2025). In order to better understand emergent patterns in responses, more studies like ours that investigate new species and utilize paired replicate designs to identify convergent patterns will be needed to uncover the factors that drive body size variation.

### Functional consequences of cranial shape associated with diet

We uncovered size-mediated and size-independent patterns of cranial shape variation that may be associated with diet since functional demands for biting and mastication can be associated with cranial shape (Marcy et al. 2024; Mitchell et al. 2024). Generally, flatter crania with longer rostra as observed in the rural phenotype are associated with reduced bite force capacity and lower mechanical efficiency (Mitchell et al. 2024; Chandler et al. 2025). Further, a dorsoventrally deeper skull, which is a substantial morphological trend observed in both the size-mediated and size-independent components of shape for urban mice (Fig. 4), provides increased reinforcement against stresses associated with jaw adduction (Mitchell et al. 2024).

Additionally, rural mice appear to have longer molar tooth rows than urban mice (Fig. 4A). Longer molar tooth rows are associated with low-quality diets and the consumption of food resources that require more chewing (Yu et al. 2017). This trend is consistent with dietary differences between habitats in our populations (Giancarli et al. 2026). Interestingly, the pattern of shorter molar tooth rows in association with urban habitats has been uncovered in the white-footed mouse [*Peromyscus leucopus* (Yu et al. 2017)] and brown rat [*Rattus norvegicus* (Puckett et al. 2020)] in New York City.

### Variation in cranial endocast volume is consistent with differences in body size

Increased cranial capacity has been posited as a response to urban structural complexity in rodents (Snell-Rood and Wick 2013). In our study, habitat did not directly contribute to variation in endocast volume. Instead, endocast volume was significantly predicted by centroid size, a proxy for body size. Interestingly, our data suggest that in urban *M. m. domesticus*, endocast volume is smaller due to reduced body size—an indirect effect of habitat. It is perhaps not surprising that our results do not support the hypothesis of increased cranial capacity in urban contexts. Evidence for the hypothesis in rodents has been limited to date (Snell-Rood and Wick 2013; DePasquale et al. 2020) and, overall, studies suggest that responses to urbanization for this trait are variable (Snell-Rood and Wick 2013; Puckett et al. 2020). Variation in response may be driven by diverse life histories and evolutionary histories in rodents (Kay and Hoekstra 2008; Fabre et al. 2012). Notably, in their original paper, Snell-Rood and Wick (2013) identified only two out of 10 species as having relatively larger cranial capacities in cities, and for neither of those did they also find body size differences among habitats. The authors also suggest that trends toward increased cranial capacity may be most prevalent in the early stages of colonization to an urban habitat (Snell-Rood and Wick 2013). House mice are human commensals that have occupied complex anthropogenic environments for thousands of years, and more recently in burgeoning pre-urban and urban settlements in both the western and eastern hemispheres (Boursot et al. 1993).

### Allometric patterns in cranial morphology

Our results uncovered a strong allometric signal of cranial shape differentiation mediated by body size. Allometry is the covariation of a trait with body size which occurs over the course of an individual’s development (*ontogenetic*) but also potentially between populations of the same species at a similar developmental stage (*static*) (Gould 1971; Cheverud 1982; Mitchell et al. 2024). The observed size-mediated shape variation (Figs. 3 and 4) indicates that urban mice occupy a distinct region of cranial morphospace along a shared allometric trajectory, reflecting differences in body size between habitats. Although there is evidence that habitat acts independently on shape variation, our results support allometry as the primary driver of divergence in cranial shape across habitat.

The observation of shape changes consistent with CREA suggests potential ecomorphological shifts. For example, there may be differences in masticatory efficiency between habitats, though assessments of other structures (i.e., mandibular morphology) and functional performance assays are needed. In our dataset, as PC1 increases, shape changes are associated with an elongation of the face/rostrum and a relative constriction of the brain case. Mean shapes (Fig. 4) also show this elongation along the anteroposterior axis in the larger-bodied rural mice and are consistent with the CREA pattern. While CREA has been established as a broad evolutionary pattern, less is known about its relevance on microevolutionary scales. Still, some comparative work has described intraspecific CREA (Schlis-Elias and Malaney 2022; Rhoda et al. 2023; Chandler et al. 2025), suggesting that the pattern is not strictly a macroevolutionary phenomenon. Importantly, the prevalence of size-related cranial shape variation in our dataset underscores the need for researchers interested in craniofacial phenotypes in the context of urbanization to consider body size as a potential confounding factor in their analyses.

It is nominally possible that we have disproportionately sampled younger mice in urban environments. While accurately assessing age in wild mice is challenging, we used hindfoot length ≥18 mm as a developmental indicator of maturity. This cut-off represents a conservative baseline within the reported adult range of 15–20 mm for the species. Because we applied this cut-off, we have interpreted these findings primarily as a signal of static allometry between urban and rural habitats. Nevertheless, mice continue to grow and develop past reproductive maturity, and we cannot rule out the possibility that the results observed here reflect some amount of ontogenetic allometry. However, this explanation is unlikely to account for the consistent patterns observed across all three cities or for the differences in body condition.

## Conclusion

Studies of morphological variation like ours are especially important for common urban taxa which are often considered pests and underrepresented in museum collections (Shultz et al. 2021; Winchell et al. 2022). Nevertheless, while phenotypic variation in wild house mice remains poorly characterized (but see Boell and Tautz 2011; Pallares et al. 2016; Parmenter et al. 2020; Ballinger and Nachman 2022; Alibert 2025), especially in cities, house mice have great potential as a vertebrate model of urban evolutionary ecology. The global distribution of house mice allows for the study of populations across a range of environmental contexts, facilitating investigation of how urbanization interacts with other factors (e.g., climate, latitude) and helping identify parallel, divergent, and idiosyncratic responses. In addition, because house mice are a genetic model system, there is a strong foundation for future genotype-phenotype and genotype-environment associations in the context of urban-associated morphological variation. For example, both body size and cranial shape are complex quantitative traits with a discrete genetic basis that has been extensively explored in both wild-derived and classical inbred strains (e.g., MacArthur 1949; Cheverud et al. 1996; Keightley et al. 1996; Kenney-Hunt et al. 2006; Wuschke et al. 2007; Chan et al. 2012; Maga et al. 2015; Pallares et al. 2015; Parmenter et al. 2016).

Our results provide strong evidence of urban-associated morphological differentiation and point to body size as a key trait mediating response to habitat. Studies, like ours, that comprehensively investigate morphology in a replicated paired urban-rural framework remain rare in vertebrates. Importantly, while the differences observed in this study could result from many factors (genetic variation, environmental variation, phenotypic plasticity, genotype-by-environment interactions, or a combination), the consistent signal across habitats makes it unlikely that patterns result from neutral processes like genetic drift. Moreover, whatever the mechanism, the consistent and meaningful differences in body size across habitat signal strong potential for impacts on fitness, motivating future studies.

## Supplementary Material

obag057_Supplemental_Files

## Data Availability

Data underlying this article are made available in its online supplementary materials. Code used for analyses and figure generation, as well as package versions can be viewed on Zenodo [https://doi.org/10.5281/zenodo.20291545]. MicroCT image data for relevant specimens have been shared to FaceBase [https://doi.org/10.25550/BE-609P].
