# Supplementary material for: Body Size and Cranial Shape Differentiation in Urban and Rural House Mice (Mus musculus domesticus)
Source: Integr Org Biol. 2026 Sep 24;8(1):obag057. doi: 10.1093/iob/obag057 (PMC13728472; doi:10.1093/iob/obag057)
Supplement: Supplementary file 1 [file obag057_supplemental_files.zip › Supplementary_Figures.docx]

**Supplementary Figure S1.** *Morphometric measurements and cranial landmark map.* **(A)** Visual summary of captured linear size metrics. All measurements are in millimeters (mm). Images are not to scale. Created in BioRender. Kupchella, S. (2026) https://BioRender.com/rqteinu. **(B)** Right lateral, **(C)** ventral, and **(D)** dorsal views of a map of the 84 landmarks used in the cranial shape analysis. Landmark descriptions are provided in S2 table.

**Supplementary Figure S2.** *Parallel Analysis Scree Plot*. Nine PCs (black dots), whose adjusted eigenvalues were >1 in a Horn’s Parallel analysis in the *paran* package, were retained.

**Supplementary Figure S3**. *Reduced allometry models and CAC/PC1 correlation.* **(A)** plotAllometry model with habitat included as an interaction term (shape ~ size * habitat). The log_centroid:habitat interaction is not significant (*P* = 0.761) indicating that urban and rural mice share an allometric slope. Panel **(B)** is the plotAllometry model with habitat included as an additive term (shape ~ size + habitat), where slopes are presumed the same and habitat is significant (*P* = 0.017), showing different intercepts. **(C)** There is a strong positive linear association between PC1 and CAC scores (Pearson’s r = 0.991), suggesting that PC1 captures the allometric relationship between size and cranial shape variation.

**Supplementary Figure S4.** *Allometric correlations.* Plots showing the relationship between size and shape variables. Panels **(A-D)** show the relationship between cranial shape PC1 and **(A)** body weight, **(B)** femur length, **(C)** body length, and **(D)** cranial endocast volume. Other panels show the positive linear association between **(E)** cranial endocast volume and cranial centroid size, and **(F)** femur length and body length. Shaded band = 95% CI of the fitted linear regression. Pearson’s correlation test was performed for each comparison. Plots are labeled with the Pearson correlation coefficient. For every test, *P* < 0.001.

**Supplementary Figure S5.** *Unedited full mean shapes with CAC, dorsal view.* A dorsal view of the full mean shapes corresponding to Figure 4C and retaining minor asymmetry. Colors differentiate habitat (orange = urban, blue = rural) and shape differences are magnified 20x. Skulls in Figure 4C were symmetrized for visual clarity.
